# Molecular and clinical characterization of new patient with 1,08 Mb deletion in 10p15.3 region

**DOI:** 10.1186/s13039-017-0336-2

**Published:** 2017-09-07

**Authors:** Anna Poluha, Joanna Bernaciak, Ilona Jaszczuk, Marta Kędzior, Beata Anna Nowakowska

**Affiliations:** 1 Department of Pediatric Hematology, Oncology and Transplantation Children’s University Hospital, Lublin, Poland; 2 Department of Medical Genetics, Cytogenetics, Institute of Mother and Child, Kasprzaka 17, A 01-211 Warsaw, Poland

**Keywords:** 10p15.3 deletion, Intellectual disability, Language impairment

## Abstract

**Background:**

Three distinct contiguous gene deletion syndromes are located at 10p chromosomal region. The deletion, involving 10p15.3 region, has been characterized by (DeScipio et al., Am J Med Genet A 158A:2152-61, 2012). However, because of the variation in size of the described deletions and lack of knowledge about the involved genes, the correlation between genotypes and patients’ phenotypes remains unknown.

**Case presentation:**

We describe female patient with de novo 1,08 Mb deletion in 10p15.3 region, similar to the patient nr seven reported by (DeScipio et al., Am J Med Genet A 158A:2152-61, 2012) but with more severe clinical features. Our patient demonstrated speech and motor delay, dysmorphic features, brain abnormalities and Tetralogy of Fallot with pulmonary atresia.

**Conclusions:**

This case shows the importance of collection of more patients with deletion in order to obtain a more precise physical map of 10p region.

## Background

Partial monosomy of 10p is a rare chromosomal aberration. Recently three distinct contiguous gene deletion syndromes, located at 10p chromosomal region, have been described. Haploinsufficiency of proximal 10p13-10p14 region, designate as DiGeorge critical region 2 (DGCR2), associated with congenital heart defect and thymus hypoplasia or T cell defect [15]. Haploinsufficiency of more distal region 10p14, responsible for hypoparathyroidism, deafness and renal anomalies (HDR Syndrome) [8, 10, 16]. And recently defined by DeScipio et al. [3] submicroscopic deletion involving 10p15.3 region, associated with intellectual disability and language impairment [3, 9, 17].

Subtelomeric deletion of 10p15.3 was up today reported in 21 unrelated patients [3, 13], two familial members of different generations [4] and one pair of monozygotic twins [17]. The first two cases were included in the large subtelomeric FISH study by Ravnan et al. [13] and the deleted region was not molecularly mapped. All other described deletions varied in size and ranged between 0,15 and 4 Mb. The size of the deletion generally does not correlate with severity of patients’ phenotype and so far critical region was not determined. However, deletion is mainly associated with cognitive/developmental and speech delay, motor delay, brain anomalies and seizures [3, 17]. Two genes, *ZMYND11* and *DIP2C*, mapping within 10p15.3 were most commonly deleted in illustrated patients [3] and were suggested to be responsible for development delay and speech impairment. Recently mutations in the *ZMYND11* gene have been demonstrated by several authors to be associated with severe speech delay and language disorder, complex cognitive, behavioral and developmental difficulties as well as dysmorphic features in some of the reported patients [1, 2, 11].

Here we present clinical and molecular data of a pediatric patient with de novo 1,08 Mb deletion in 10p15.3 region and clinical features suggestive of del22q11. Our patient has similar deletion size to the patient nr 7 reported by DeScipio et al. [3], but more severe clinical phenotype, including brain malformation, and heart abnormalities observed only in 2/21 patients with 10p15.3 deletion (Table 1).

**Table 1:** Summary of clinical features in the cohort of Vargiami et al. [17] and DeScipio et al. [3], with distinction of patient nr 7, patient with missense mutation in ZMYND11 gene [1] and our index patient

	DeScipio et al., Varigiami et al.	Patient 7 from DeScipio et al. cohort with the closest overlapping deletion	Patient with mutation in *ZMYND11* gene described by Cobben et al.	Patient with mutation in *ZMYND11* gene described by Moskowitz et al.	Our patient
sex	Male:Female 8:6	Male	Male	Female	Female
Age at report	1y9m – 48y	5y	7y	24y	5y
Birth weight	< 3rd centile – 25th centile	3402 g (25th centile)	1867 g (<3rd centile)	3740 g (50th centile)	3370 g (25th centile)
Height	<3rd centile – 75th centile	25th centile	< 3rd centile	Not provided	< 3rd centile
Weight	<3rd centile – > 95th centile	75th centile	Not provided	Not provided	< 3rd centile
Head circumference	10th - >95th centile	75th centile	Microcephaly (<3rd centile)	Microcephaly (<3rd centile)	25th centile
Cognitive/behavioral/developmental differences	13/13	Hyperactivity	Severe developmental delay	Severe global developmental delay, able to understand to some degree and can communicate using a few sings, happy disposition, smiles almost constantly	Mild ID, temper tantrums, aggression
Speech delay	10/10	Severe One or two word sentences at 5y	At the age of 7 years uses only two words	Non verbal, makes sounds	Severe Single words at 5y
Motor delay	11/11	Started to walk independently at 2,5y Unable to run well or ride a bicycle at 5y	At the age of 7 years walks behind a walking device	Severe motor delay, ataxic, wide base gait, walks only short distances with assistance.	Started to walk independently at 2y Clumsy running at 5y
Craniofacial dysmorphic features	9/12 Inconsistent	Plagiocephaly, hypertelorism, prominent columella, hypoplastic alae,	Slight metopic ridge, low-set ears, hypopigmentation of the right eyebrow and eyelashes, telecanthi, epicanthic folds, slant-up and narrowing of palpebral fissures, broad nasal bridge, small nares with broad alaenasi, smooth philtrum with thin upper lip and everted lower lip, widely-spaced teeth, retrognathia.	Bulbous nose with wide base and ridge,, deep set eyes, long palpebral fissures, epicanthal fold, prominent jaw	Flat face, mild synophrys, long eyelashes, long palpebral fissures, epicanthal folds, wide nasal ridge, low set, posteriorly rotated and slightly protruding ears, underdeveloped antitargus, short chin
Brain abnormalities	6/8 cortical atrophy (4), hydrocephalus (1), arachnoid cyst (1)	No	Delayed myelination	Cerebral atrophy and delayed myelination without evidence of focal abnormality	Chiari malformation type I with spinal cord edema requiring surgical decompression
Hypotonia	7/13	No	Yes	Yes	in infancy
Hand/ft anomalies	5/13	5th finger clinodactyly, fetal pads, pes planus, clinodactyly of toes 3,4,5	Valgus feet, short metacarpals of both hands and a brachymesophalanx V, broad hands with deep palmar creases and pillowing of the areas between the grooves, short tapering fingers and broad feet with short toes and small nails	5th finger clinodactyly, small feet	5th finger clinodactyly pes planus
Seizures	3/9	Partial complex	Absence seizures	Severe intractable seizures	Absence seizures
Cardiac anomalies	2/11 bicuspid aortic valve, patent foramen ovale, murmur	Systolic murmur	Spontaneously closed VSD	None	Tetralogy of Fallot with pulmonary atresia
Other features		Diaphragmatic hernia, mildly narrow palate, shawl scrotum and wide-based gait	Feeding difficulties requiring supplementary tube feeding, fusion of the 2nd and 3rd vertebrae and compression of the myelum, pyloric stenosis non-descended testes and urethral stenosis, inverted and widely-spaced nipples, dimples on elbows and knees	Neurogenic bladder Severe eosinophilic gastroesophagitis, gastroesophageal reflux Asthma, multiple food, environmental and medication allergies Myopia Bilateral moderate hearing impairment (family history positive for hearing impairment)	wide–base gait protein C deficiency

### Patient

Female patient was born as a third child of a healthy nonconsanguineous couple in a family without a history of congenital malformation nor intellectual disability. The pregnancy was uneventful with no confirmed teratogenic exposure and full term. Girl was born with weight 3370 g, length – 55 cm and OFC - 35 cm initially in a good condition which started to deteriorate rapidly due to severe congenital heart defect - tetralogy of Fallot with pulmonary atresia. Single stage cardiosurgery has been performed on 7th day after delivery. The postoperative period has been complicated by thrombotic events due to protein C deficiency requiring surgical clot removal. Following her initial neurologic examination at the age of 3,5 months which revealed axial hypotonia and head circumference of 38,5 cm (10th centile) with normal results of cranial ultrasound, she has been systematically evaluated by pediatric neurologist. Her developmental milestones were markedly delayed. She started to walk unaided at 24 months and she did not vocalize till 3 years of age. The results of her neuropsychologic evaluation at this time indicated mild mental retardation with difficulties in gross motor skills and socioemotional functions with relatively well visuomotor skills. At 3 years of age her parents noticed unsteady, wide-base gait and unusual behavior presenting as unprovoked temper tantrums, aggression and sudden-onset interruption of on-going activities with blank stare and impairment of consciousness. Abnormal EEG (Electroencephalography) results at that time indicated epilepsy and the anti-epileptic drugs has been introduced. Additionally her brain MRI (Magnetic resonance imaging) revealed downward displacement of medullary tonsils (22 mm below foramen magnum) with spinal cord edema without syrinx, consistent with Chiari malformation type I. She underwent the posterior fossa decompression with C1 and partial C2 laminectomy accompanied by duraplasty which later required two additional surgical corrections. After last surgery, significant progression of her development was noted.

**Fig. 1:**
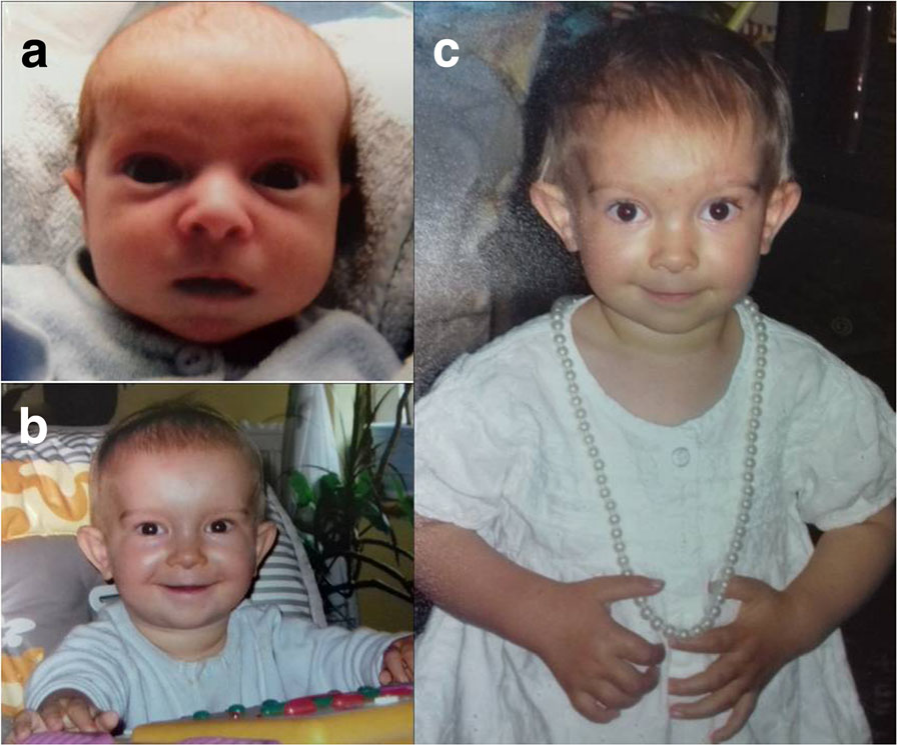
**a** Patient on the first day of life: round face, long palpebral fissures, epicanthal folds, wide nasal root and wide nasal bridge. **b** Patient at the age 8 months: round face, upstanding palpebral fissures, low set nasal root, short columella, narrow upper lip, long and protruding ears. **c** Patient at the age 2 years: frontal bossing, ocular hypertelorism, long upstanding palpebral fissures, long lashes, low set nasal root, wide nasal bridge, narrow upper lip, short chin

Girl was initially assessed at our Genetic Clinic at the age of three. She presented with developmental delay, mild mental retardation, severe language impairment and failure to thrive. Her height was 89 cm(< 3rd centile), weight was 12 kg(< 3rd centile) and her head circumference was 48,5 cm (25th centile). Dysmorphic features included: flat face, mild synophrys, long eyelashes, long palpebral fissures (2,8 cm >90th centile), epicanthal folds, wide nasal bridge, low set, posteriorly rotated and slightly protruding ears with underdeveloped antitargus, short chin and fifth finger clinodactyly. Dysmorphic features in combination with, short stature, developmental delay, impaired speech development and congenital heart defect were suggestive of DiGeorge syndrome (Fig. 1a–c).

## Methods

Genetic diagnostic studies were done including chromosome analysis after GTG banding with a resolution of approximately 500 bands per haploid genome.

Genomic DNA was extracted from patient’s peripheral blood cells using a Genomic DNA purification kit (Puregene, Gentra Systems, Minneapolis, MN) according to the manufacturer’s instruction.

MLPA (245 SALSA MLPA probemixes, MRC-Holland) analysis was performed to exclude the 22q11 deletion.

Array CGH was performed using a 180 K oligonucleotide microarray (CytoSure, ISCA v2, Oxford Gene Technology, Oxford, UK). DNA of the patient was hybridized against a female control. Labeling and hybridization were performed following the manufacturer’s protocols (Invitrogene, BioPrime Array CGH, Carlsbad, CA). Briefly, 1 μg of DNA was labeled overnight by random primers. Labeled products were purified on the columns centrifugal filters (Invitrogene, BioPrime Array CGH) according to the manufacturer’s instruction. After probe denaturation and prehybridization with Cot-1 DNA, hybridization was performed at 65 °C with rotation for 72 h. After washing the array was analyzed with the Agilent scanner and Feature Extraction software (Agilent Technologies, Santa Clara, CA) and text file outputs from the quantization analysis were imported to CytoSure Interpret Software (Oxford Gene Technology) for copy number analysis.

FISH analyses of the 10p15.3 region was performed according to a standard protocol, using BAC clone RP11–62O22. Briefly, a 500 ng DNA of BAC clone was labeled with Spectrum Red dUTP by random prime method (Invitrogene, BioPrime Array CGH), according to the manufacturer’s’ protocol. Slides were viewed on a Zeiss Axioplan2 fluorescence microscope and images were captured and analyzed using Applied Spectral Imaging Acquisition 5.0 analysis system (Applied Spectral Imaging, Inc. Vista, CA).

## Results

Chromosome analysis revealed normal female karyotype. Also MLPA analysis for common deletions was normal.

**Fig. 2:**
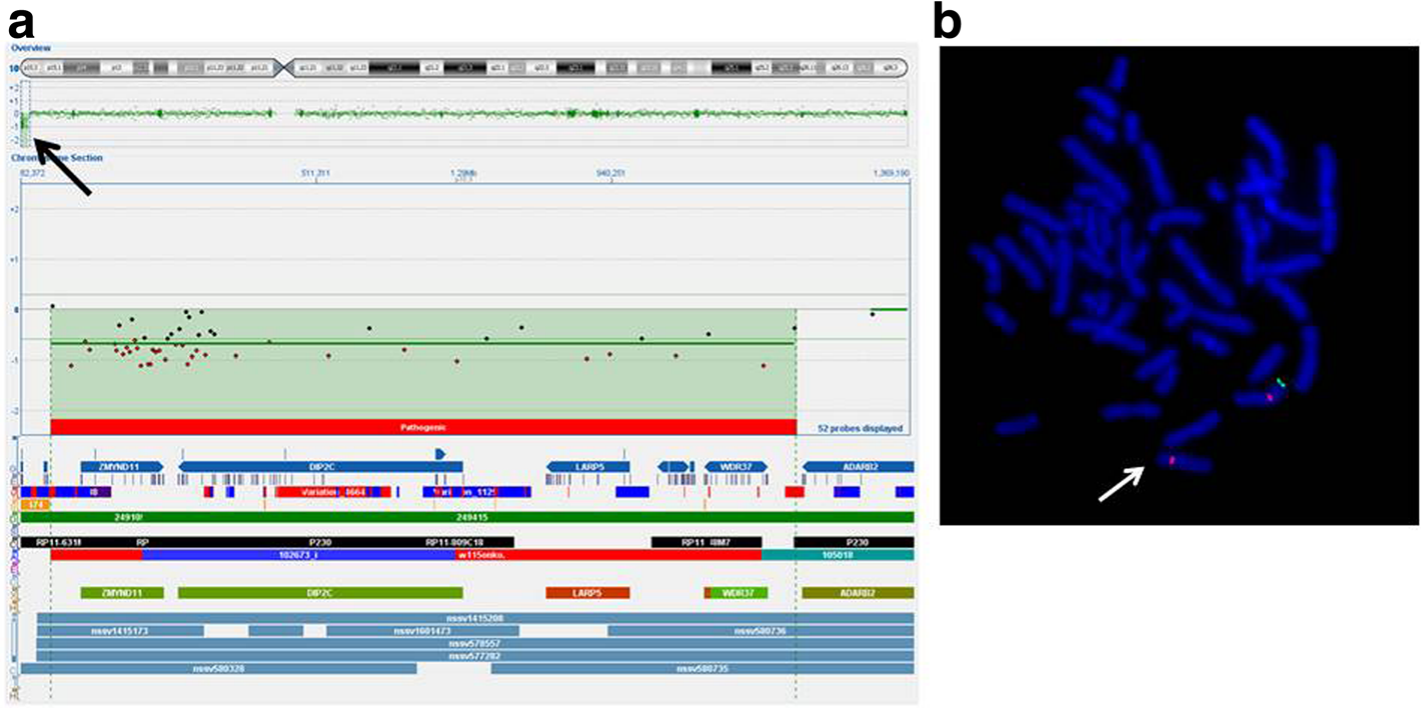
**a** Array-CGH analysis showing deletion of the 10p15.3 region. **b** FISH analysis of the 10p15.3 region using BAC clone RP11–62O22 (green), confirmed deletion

The whole genome CGH array identified a 1,08 Mb deletion on chromosome 10p15.3 (Fig. 2a). The proximal breakpoint was mapped at the position 126,145 and the distal breakpoint at 1,204,340 (UCSC Genome Browser on Human, hg18). No other CNVs have been detected.

FISH analysis with BAC clone RP11–62O22 confirmed the deletion (Fig. 2b) and parental studies showed that deletion occurred de novo*.*

Deleted genomic region harbors 8 RefSeq known genes; *ZMYND11, DIP2C, RRR26, LARP4B, GTPBP4, IDI2, IDI1, WDR37*. Only 4 OMIM annotated; *ZMYND11, DIP2C, IDI1*, *IDI2,* and two dosage sensitive; *LARP4B (LARP5)* and *IDI1*. Happloinsufficiency score of the dosage sensitive gene is 0,601 and 0,488 for *LARP4B* and *IDI1*, respectively [5].

## Discussion

We report female patient with 1,08 Mb deletion on chromosome 10p15.3 presenting with development delay, severe language impairment, motor delay, dysmorphic features, hypotonia, seizures, brain malformations and severe congenital heart disease. To our knowledge 25 patients has been reported so far with 10p15.3 deletion and some overlap in phenotypic features. Though not for all described cases detailed clinical information is known, the patients’ phenotype does not simply correlate with the size of the deletion. Our patient has similar deletion to the patient seven from DeScipio et al., [3] study, but more severe phenotype, including congenital heart condition and Chiari malformation type 1 not seen before in this patients’ cohort. Cardiac anomalies have been observed only in 2 out of 25 patients with this deletion. As for the brain abnormalities, they were noted in four out of six radiologically evaluated patients reported by DeScipio et al. [3]: hydrocephalus (1 patient), small arachnoid cyst (1 patient) and cortical atrophy (2 patients). The later has also been present in the female twins reported by Vargiami et al. [17]. None of the previously described patients has been found to have structural defects of the cerebellum. It should be noted that the patient reported by DeScipio et al. [3] is a male while our patient is a female. However limited information about the deleted genes’ function does not allow to determine if sex factor could contribute to the severity of our patient’s clinical symptoms. None such correlation has been pointed out in the cohort of patients reported by DeScipio et al. [3] in which female to male ratio was 10:9.

Little is currently known about the genes located within 10p15.3 region, and this complicates the genotype – phenotype correlation. DeScipio et al. [3] distinguished two genes, *ZMYND11* and *DIP2C*, although no single gene was deleted in all 19 studied individuals. Several cases with a de novo mutation in *ZMYND11* gene have been reported [1, 2, 6, 11]. First case with a G > A substitution in codon 239, predicted to alter a splice site in the *ZMYND11* gene [6]. However, this patient had an autism spectrum disorder but no intellectual disability and no obvious dysmorphism. Second patient with a de novo missense mutation C > T in codon 1798 in *ZMYND11* gene presented severe developmental delay and dysmorphic feature (Table 1). This variant changed an evolutionary highly conserved, positively charged, arginine into a neutral tryptophan [1]. Coe and co-authors (2014) in their study of large cohort of patients with neurodevelopmental diseases, using integrated analysis of copy number variants and single-nucleotide variants followed by resequencing of candidate genes, identified five different truncating *ZMYND11* mutations in patients with overlapping clinical presentation including speech and motor delay, borderline IQ, mild dysmorhism as well as complex behavioral and developmental problems. They also suggested that truncating mutations in *ZMYND11* gene are likely to be associated with other more complex neuropsychiatric disorders. More recently Moskowitz et al. [11] presented a female patient with a severe global developmental delay, intractable epilepsy, hypotonia and dysmorphic features associated with a de novo missense mutation in *ZMYND11* gene.

*ZMYND11* (OMIM 608668) is located to the nucleus and regulates RNA polymerase II elongation [19]. *DIP2C* (OMIM 611380) is expressed in all adult and fetal tissues, including specific adult brain regions, but except lung and pancreas, where expression was detected at low level [12]. However two different genes are deleted in our patient and are known to be dosage sensitive: *LARP4B* and *IDI1. LARP4B* is not annotated in OMIM and still very little is known about its function. It belongs to an evolutionarily conserved family of factors with predicted roles in RNA metabolism. Schäffler et al. [14] demonstrated its role in bridging mRNA factors of the 3′end with initiating ribosomes. Overexpression of LARP4B stimulated protein synthesis, whereas knockdown of the factor by RNA interference impaired translation of a large number of cellular mRNAs. Additionally, Wang et al. [18] suggested that abnormal expression of Larp4b can be found in leukemia patients. *IDI1* gene (OMIM 604055) catalyzes a critical activation step in isoprenoid pathway and has a reduced activity in liver tissue from patients with the peroxisomal deficiency diseases Zellweger syndrome and neonatal adrenoleukodystrophy [7]. Based on this very limited information about the genes function it is very difficult to draw conclusion which of these genes can be crucial for observed phenotypes and how they can influence the variability in clinical features. Also, so far no patients with mutation in other genes than *ZMYND11* have been described. However, common clinical features observed in most patients with deletion of 10p15.3 and patients with mutations in *ZMYND11* suggest that haploinsufficiency of *ZMYND11* contributes to the clinical features of 10p15.3 deletions syndrome and most likely it is responsible for intellectual disability in those patients. But molecular and clinical description of new patients with deletion in 10p15 is necessary before the full gene – phenotype correlation will be established for this region.
